# Co-seeding grasses and forbs supports restoration of species-rich grasslands and improves weed control in ex-arable land

**DOI:** 10.1038/s41598-022-25837-4

**Published:** 2022-12-08

**Authors:** Réka Kiss, Balázs Deák, Katalin Tóth, Katalin Lukács, Zoltán Rádai, András Kelemen, Tamás Miglécz, Ágnes Tóth, Laura Godó, Orsolya Valkó

**Affiliations:** 1 ‘Lendület’ Seed Ecology Research Group, Institute of Ecology and Botany, Centre for Ecological Research, Alkotmány str. 2–4, Vácrátót, 2163 Hungary; 2 Department of Ecology, University of Debrecen, Egyetem sqr. 1, Debrecen, 4032 Hungary; 3 Juhász-Nagy Pál Doctoral School, University of Debrecen, Egyetem sqr. 1, Debrecen, 4032 Hungary; 4 Centre for Ecological Research, National Laboratory for Health Security, Karolina út 29, Budapest, 1113 Hungary; 5 Department of Ecology, University of Szeged, Közép Fasor 52, Szeged, 6726 Hungary; 6 Hungarian Research Institute for Organic Agriculture, Miklós tér 1, Budapest, 1033 Hungary

**Keywords:** Ecology, Grassland ecology, Restoration ecology

## Abstract

Sowing is widely used for the restoration of species-rich grasslands but still there are knowledge gaps regarding the most suitable application of different seed mixtures. We tested the effect of seed mixtures application timing on the establishment of sown forbs and weed control. 36 experimental plots with nine sowing treatments were established in an abandoned cropland in Hungary. Grass-seeds, diverse forb seed mixture and the combination of the two were applied: diverse forb mixture was sown simultaneously or 1, 2 or 3 years after grass sowing, in plots sown previously with grass or in empty plots (fallows). All sowing treatments supported the rapid establishment of the sown species in large cover and hampered weed encroachment. Forbs performed better when sown into fallows than in grass-matrix and forbs establishment was worse in older fallows than in younger ones. Grasses expressed a strong priority effect, especially when forbs were sown at least two years later than grasses. We also investigated the relation between seed germinability, weather parameters and establishment success. Germination rate in the greenhouse could not predict the establishment success of forbs in the field and showed great differences between years, hence we recommend sowing target forbs in multiple years.

## Introduction

Halting degradation of terrestrial ecosystems by various restoration measures is in the focus of the United Nations Decade of Ecosystem Restoration (2021–2030). Countries all over the world should contribute by rehabilitating and restoring degraded ecosystems, including grasslands, to prevent or at least mitigate the catastrophic consequences of climate change and loss of biodiversity^1^.

Grasslands are among the most species-rich ecosystems in the word^2^ and can be substantial carbon sinks in the changing climate^1,3^. They also provide important ecosystem services such as water supply and regulation, soil protection, pollination, biomass production and cultural services^4^. The diversity of grasslands is positively related to their stability and resistance against disturbances and to the ecosystem services provided^5–7^. Halting their degradation, decreasing their fragmentation and increasing their area, as well as conserving their diversity are as important as afforestation principles^8,9^. By restoring species-rich grasslands not only plant diversity can be increased, but also habitat heterogeneity, which in turn facilitates restoration of other life forms^10^. These targets can be achieved both by restoration of degraded species-rich grassland ecosystems^11^ and by grassland recreation in abandoned croplands^12,13^.

The abandonment of croplands is a global phenomenon^14,15^ that is especially typical in the Northern Hemisphere^16^. In the European Union 11% of all croplands is considered to have high likeliness to be abandoned in the period of 2015–2030^17^. Besides socio-economic changes, the main reasons of abandonment are the inadequate edaphic and climatic conditions for crop production^15,18–20^.

The low level of abiotic (i.e., high nutrient availability) and biotic (i.e., low competitive pressure) filtering in abandoned croplands allows the establishment of weedy vegetation from the weed-contaminated soil seed bank^21–23^. Weed encroachment provides ecosystem disservices that can also affect the neighbouring natural habitats and agricultural fields. In abandoned croplands active restoration methods are needed to suppress weeds, control vegetation development and achieve a successful grassland recovery^24,25^. For rehabilitating and restoring grassland ecosystems in a cost-effective way at the envisaged global scale, fine-tuning of already known restoration methods is necessary.

Seed sowing is a widely used method that offers predictable results which can be implemented at various scales. Sowing propagules of matrix grasses and forbs proved to be a feasible tool for restoring diverse grassland habitats and to suppress weeds^24,26^. The composition of seed mixtures differs considerably regarding the aim, budget, machinery and timeframe available for restoration^27^. Sowing the propagules of a single grass species or low-diversity grass seed mixtures is effective when the aim is the fast restoration of the grass matrix^28,29^. The closed sward of grass species suppresses weeds, decreases soil openness and therefore the soil erosion^30,31^. Sowing only a few grasses is a cost-effective measure, as seeds of a limited number of species (usually 1–8 grass species) should be easily purchased or collected^27^. The costs of acquiring diverse grass-forb seed mixtures are considerably higher^27,32^ and it is often difficult to obtain the seeds of a large set of species in sufficient quality and quantity. That is one of the reasons why diverse seed mixture sowing is seldom applied in large-scale restoration projects. Grass sowing is widely used approach in large-scale restoration projects, but it should be considered that the developed closed sward not only suppresses weeds but also hampers the establishment of late-successional target grassland forbs^24,29,33^. Application of diverse seed mixtures by overseeding the grass matrix^34,35^ or sowing into gaps with bare soil^24,36^ is used mainly to increase species richness of species-poor restored grasslands.

The order of species arrival is a major driver of the species composition of the developed grasslands. Previous studies found that a priority effect can be detected even when species are sown with only three weeks difference^37,38^. Studies suggest that species arriving first tend to dominate the recovering vegetation^39,40^. If grasses are sown first, the establishment of other functional groups is generally negatively affected. In contrast, in the case of forb sowing, priority effect is missing or weaker compared to grass sowing^33,39,41^. The order and timing of sowing different components of the seed mixtures have a long-term effect on the vegetation, that can be detected also in later successional stages^33^.

**Figure 1:**
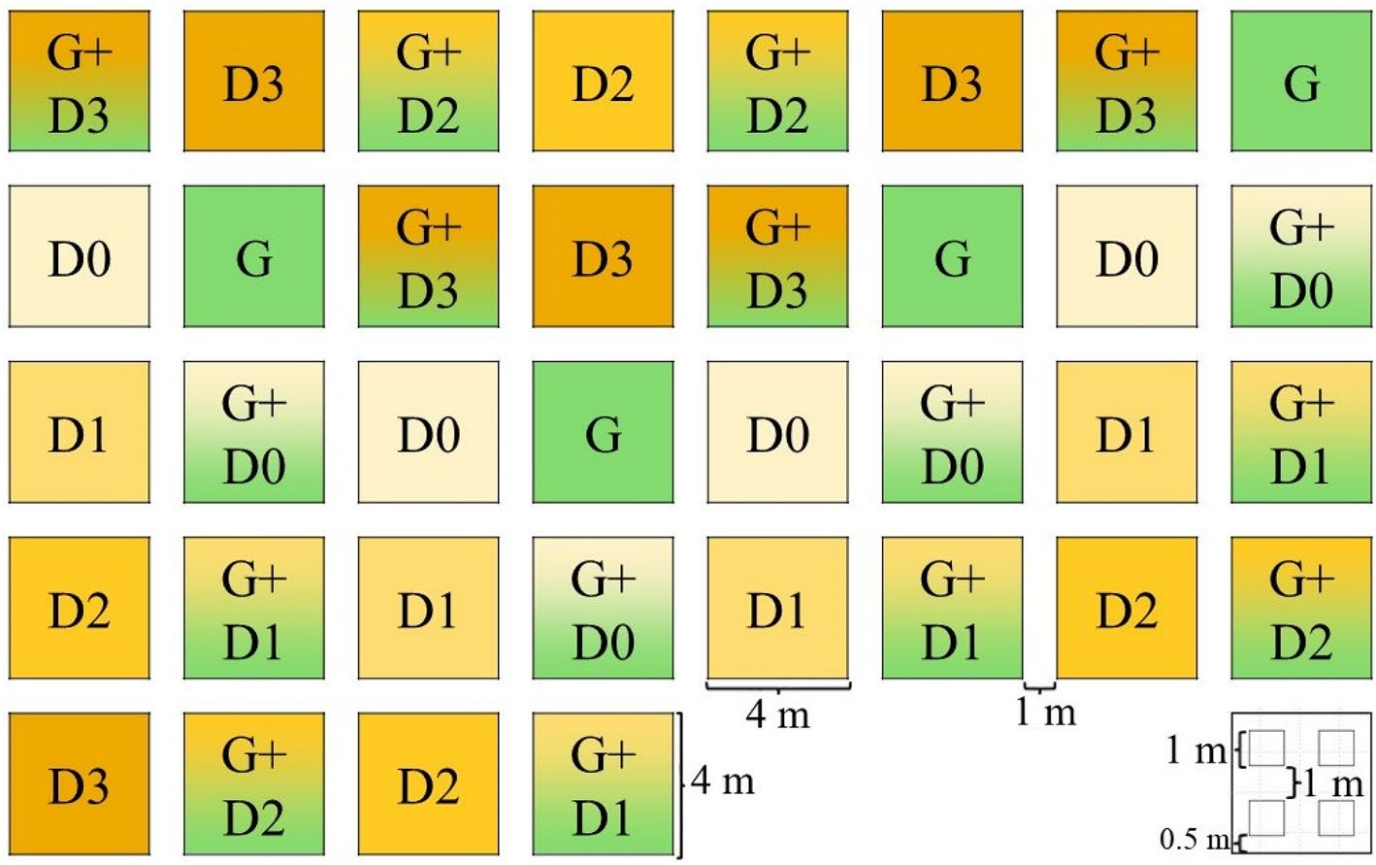
Design of the experimental site. Abbreviations indicate treatment types: G + D—plots sown with grass and diverse forb seed mixture; D—plots sown only with diverse forb seed mixture; G—plots sown with grass (*F. pseudovina*) seeds. Numbers indicate the age of the grass-matrix or fallow when diverse forb seed mixture (D) was added. Bottom-right square indicates the location of the permanent 1 m × 1 m subplots within the plots.

Understanding the mechanism of vegetation recovery and post-restoration assembly rules can enhance restoration success. In our study, we established a mesocosm experiment, where we manipulated the initial floristic composition of the experimental plots by sowing seed mixtures with various diversity and timing (in total nine treatments with four replicates, Fig. 1, Table 1). Plots received only grass seeds (*Festuca pseudovina*) (G plots), only diverse forb seed mixture (D plots) or the combination of both (G + D plots). Forb mixtures were sown into two types of plots: plots sown with grass seeds (G + D plots) or fallows (D plots). Forbs were sown 0, 1, 2 or 3 years after grass seed sowing (G + D0, G + D1, G + D2, G + D3 plots) or after leaving the plot fallow for 0, 1, 2 or 3 years (D0, D1, D2, D3 plots). This study design enabled us to test the effect of grass matrix age and fallow age on the establishment success of forbs under field conditions and give recommendations for upcoming restoration projects. In addition, we conducted a germination test under greenhouse conditions to detect differences in germination rate of the sown forbs between sowing years and linked our results to weather parameters of the seed ripening period. We also studied the relation between the seed mass and establishment success of the sown forbs under field conditions. Our main goal was to identify the most feasible timing of seed sowing and the composition of seed mixtures to support restoration practitioners in creating diverse grassland communities, enabling the establishment of target forb species and the suppression of weeds, thus supporting the development of species-rich communities.

**Table 1:**
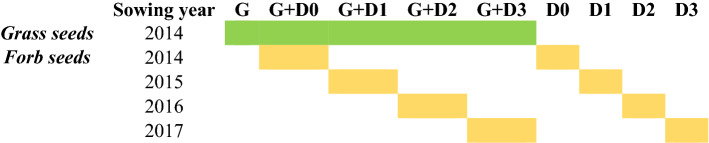
Experimental design.

First column indicates seed-mixture type, second column indicates the sowing year of grass (G) and diverse forb seed mixture (D). First row indicates treatment types. Colours indicate the seeds sown: green—grass (*F. pseudovina*) seeds; yellow—diverse forb seed mixture.

We hypothesised that (i) Forbs can establish better when sown on fallow than when sown into the grass matrix; (ii) Both sown grasses and forbs hamper the establishment of weeds; (iii) Time since cropland abandonment (fallow age) or grass sowing (grass matrix age) negatively affect the establishment success of sown forbs. As weather conditions during the seed ripening period affect seed quality^42^ we expected differences in seed germinability of sown forbs between sowing years.

## Results

### Vegetation development

**Figure 2:**
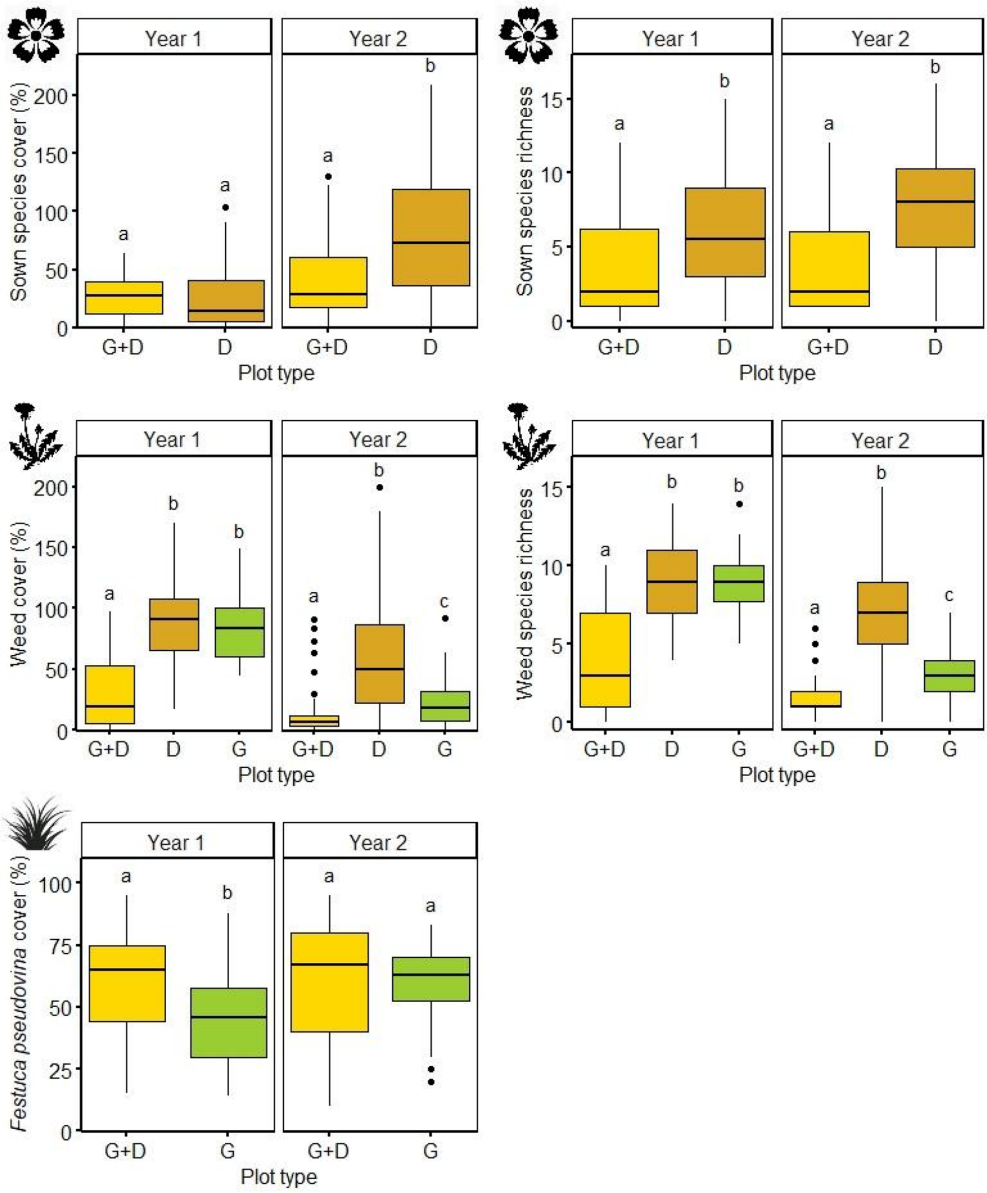
Vegetation development in different treatments in the first and second study years. Lower-case letters indicate significant differences between treatments (comparison with emmeans, p ≤ 0.05). Boxplot lines represent median values. *G + D*—plots sown with grass and diverse forb seed mixture, *D*—plots sown only with diverse forb seed mixture, *G*—plots sown with grass (*F. pseudovina*) seeds.

Out of the 20 sown forb species 19 were able to establish in at least one of the experimental plots. In both the G + D and D plots the cover of sown forbs increased from the first year to the second (Table 2, Supplementary Table S1, Supplementary Fig. S1). Both cover and species richness were significantly lower in G + D plots than in D plots in both years (Table 3, Fig. 2). The cover and species richness of weeds decreased generally from the first to the second year, with the exception G + D3 plots, where the cover was higher in the second year. Both scores were the lowest in G + D plots and highest in D plots. The cover and species richness of weeds was similar in D and G plots in the first study year, but became significantly different in the second study year, when both variables were significantly lower in G plots. The cover of *F. pseudovina* did not change over the years and was higher in G + D plots than in G plots, but only in the first study year.

**Table 2:** Effect of study year, grass-matrix and fallow-age and their interaction on the cover and richness of species groups (GLM).

Sowing treatment	Sown forbs				Weed				*Festuca pseudovina*	
	Cover (%)		Richness		Cover (%)		Richness		Cover (%)	
	z	p	z	p	z	p	z	p	z	p
**Study year 2**										
D	4.601	** < 0.001**	1.791	0.073	− 4.171	** < 0.001**	− 3.222	**0.001**		
G + D	2.940	**0.003**	− 0.067	0.948	− 5.735	** < 0.001**	− 6.400	** < 0.001**	− 0.551	0.582
G					− 9.146	** < 0.001**	− 10.836	** < 0.001**		
**Grass-matrix age**										
G + D1	− 1.999	**0.046**	− 1.537	0.124	− 2.797	**0.005**	− 1.668	0.095	0.505	0.614
G + D2	− 1.297	0.195	− 7.281	** < 0.001**	− 6.095	** < 0.001**	− 7.220	** < 0.001**	2.505	**0.012**
G + D3	− 3.451	** < 0.001**	− 7.281	** < 0.001**	− 5.674	** < 0.001**	− 7.045	** < 0.001**	2.356	**0.019**
**Fallow age**										
D1	2.461	**0.014**	3.279	**0.001**	− 2.463	**0.014**	− 1.094	0.274		
D2	− 6.449	** < 0.001**	− 3.100	**0.002**	0.323	0.747	− 1.514	0.130		
D3	− 9.601	** < 0.001**	− 5.807	** < 0.001**	− 0.423	0.672	− 1.636	0.102		
**Study year 2 × age of grass matrix**										
G + D1	0.286	0.775	0.100	0.921	0.225	0.822	0.216	0.829	0.157	0.876
G + D2	− 3.564	** < 0.001**	0.334	0.738	2.948	**0.003**	3.885	** < 0.001**	0.894	0.372
G + D3	− 1.729	0.084	0.625	0.532	5.874	** < 0.001**	4.752	** < 0.001**	0.361	0.718
**Study year 2 × age of fallow**										
D1	− 0.545	0.586	− 1.050	0.294	− 0.889	0.374	− 2.432	**0.015**		
D2	1.979	**0.048**	0.907	0.364	0.879	0.380	1.936	0.053		
D3	2.816	**0.005**	0.081	0.936	4.169	** < 0.001**	2.692	**0.007**		

Significant effects are marked in boldface (p < 0.05). G + D-plots sown with grass and diverse forb seed mixture; D-plots sown only with diverse forb seed mixture; numbers indicate the age of the grass-matrix (G + D plots) or fallow-age (D plots) when diverse forb seed mixture was sown in the plots.

**Table 3:** Overall (the two study years pooled together) and by study year (the two study years separately) differences of species groups cover and richness between the treatments (factor-level comparisons with emmeans).

	Sown forbs				Weed				*Festuca pseudovina*	
	Cover (%)		Richness		Cover (%)		Richness		Cover (%)	
	t	p	t	p	t	p	t	p	t	p
**Overall**										
G + D – D	− 4.140	** < 0.001**	− 8.413	** < 0.001**	− 10.018	** < 0.001**	− 13.110	** < 0.001**		
G + D – G					− 6.522	** < 0.001**	− 8.733	** < 0.001**	2.791	**0.015**
D – G					2.753	**0.017**	4.106	** < 0.001**		
**Year 1**										
G + D – D	− 0.277	0.959	− 5.049	** < 0.001**	− 6.678	** < 0.001**	− 10.229	** < 0.001**		
G + D – G					− 5.780	** < 0.001**	− 9.766	** < 0.001**	4.000	** < 0.001**
D – G					0.403	0.914	− 0.010	1.000		
**Year 2**										
G + D – D	− 4.717	** < 0.001**	− 6.892	** < 0.001**	− 9.646	** < 0.001**	− 11.831	** < 0.001**		
G + D – G					− 2.970	**0.009**	− 4.421	** < 0.001**	0.321	0.945
D – G					5.961	** < 0.001**	7.440	** < 0.001**		

Significant differences are marked in boldface (p < 0.05). G + D—plots sown with grass and diverse forb seed mixture; D—diverse forb seed mixture.

### Effect of grass matrix- and fallow age

**Figure 3:**
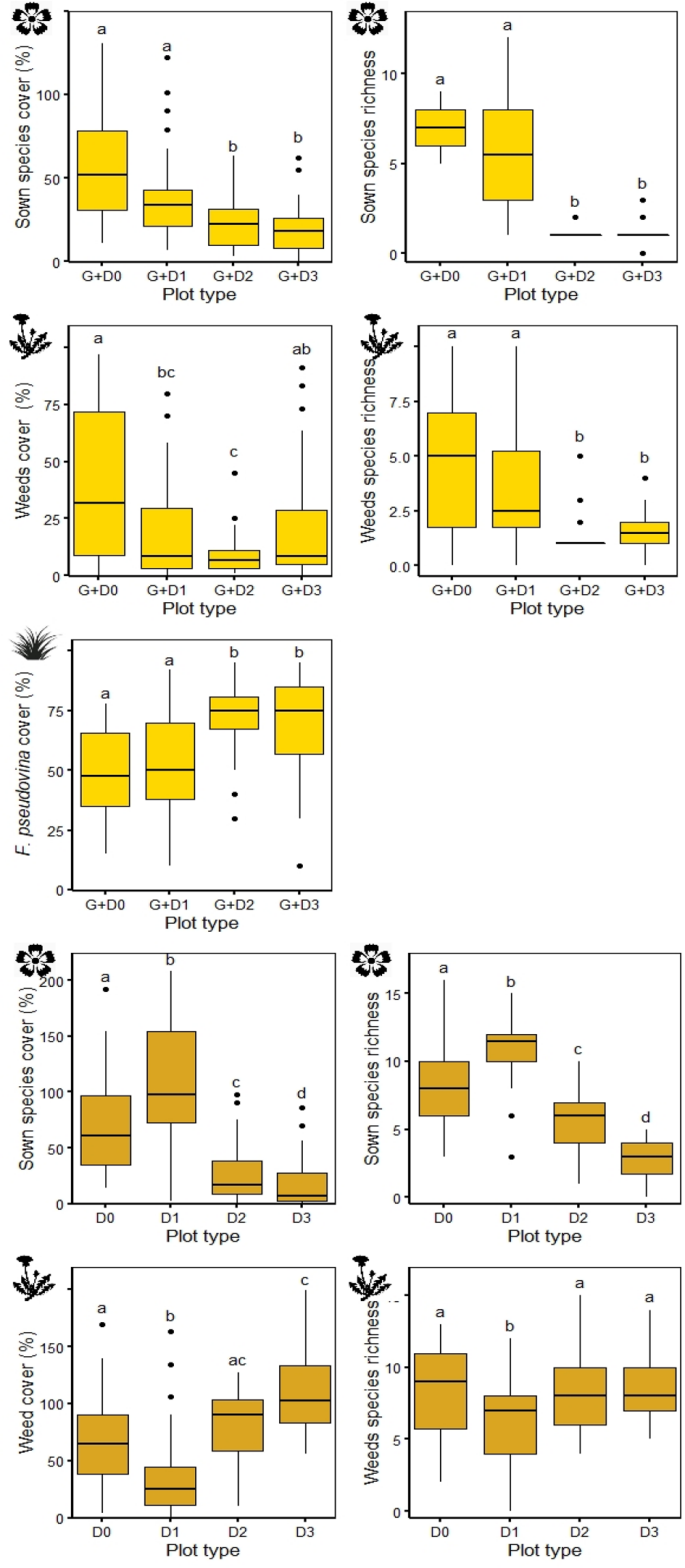
Grass-matrix age and fallow-age effect on species groups pooled for the two study years. Lower-case letters indicate significant differences (comparison with emmeans, p ≤ 0.05). Boxplot lines represent median values. *G + D*—plots sown with grass and diverse forb seed mixture, *D*—plots sown only with diverse forb seed mixture, *G*—plots sown with grass (*F. pseudovina*) seeds. Numbers indicate the age of the grass-matrix or fallows when diverse forb seed mixture (D) was added.

Age of grass-matrix and fallow (Table 2, Fig. 3) had mostly negative effects on the studied variables. In G + D plots cover and species richness of sown forbs decreased with increasing time lag between grass and D seed mixture sowing. Species richness of sown forbs decreased markedly if sown in grass-matrixes older than one year (G + D2 and G + D3). This trend was more pronounced in the second study year (Supplementary Table S2, Supplementary Fig. S2). Increasing age of grass-matrix also resulted in lower cover and species richness of weeds, especially in the first study year, while to the second study year weed cover increased significantly in G + D3 plots. The cover of *F. pseudovina* increased with age of grass matrix, the difference being significant only in the second study year. In D plots both sown species cover and richness were the highest in D1 plots, when sowing happened in one-year-old fallows (Supplementary Table S2, Supplementary Fig. S2). In older fallows both cover and species richness of sown forbs decreased significantly. The trend was detectable in both study years, although not always statistically significant. Weed cover and richness was lowest in the D1 plots. The effect of fallow age on weed cover was more pronounced in the second study year.

### Four-year vegetation development in early sown plots

We analysed the four-year long datasets of G + D0, G + D1, D0 and D1 plots. Sown forbs cover had a non-significant tendency to increasing over the years (Table 4, Supplementary Table S3, Supplementary Fig. S3), but their richness was similar across the years in both G + D and D plots. In the first three study years, although not always significantly, the cover of sown forbs was higher in G + D0 plots than in G + D1 plots; it was lower only in the fourth study year (Supplementary Table S4, Supplementary Fig. S4). There was no difference in sown forbs richness between G + D treatment plots. In D plots we detected a higher cover and species richness of sown forbs in almost all years in D1 plots than in D0 plots.

**Table 4:** Effect of treatment and study year on the cover and richness of species groups in the four years of the early sowing plots (GLM).

Sowing treatment	Sown forbs				Weed				*Festuca pseudovina*	
	Cover (%)		Richness		Cover (%)		Richness		Cover (%)	
	z	p	z	p	z	p	z	p	z	p
**Delayed sowing**										
G + D	− 2.287	**0.022**	− 1.537	0.124	− 3.230	**0.001**	− 1.668	0.095	0.361	0.718
D	3.852	** < 0.001**	3.279	**0.001**	− 1.538	0.124	− 1.094	0.274		
**Study year**										
G + D										
2	3.320	** < 0.001**	− 0.067	0.947	− 6.622	** < 0.001**	− 6.400	** < 0.001**	− 0.394	0.694
3	4.135	** < 0.001**	− 0.890	0.373	− 11.880	** < 0.001**	− 7.271	** < 0.001**	− 1.340	0.180
4	3.504	** < 0.001**	− 0.820	0.412	− 9.981	** < 0.001**	− 6.982	** < 0.001**	0.138	0.890
D										
2	7.175	** < 0.001**	1.791	0.073	− 2.605	**0.009**	− 3.222	**0.001**		
3	8.128	** < 0.001**	0.768	0.443	− 4.282	** < 0.001**	− 7.316	** < 0.001**		
4	7.838	** < 0.001**	0.453	0.651	− 3.785	** < 0.001**	− 7.966	** < 0.001**		
**Delayed sowing × study year**										
G + D										
2	0.348	0.728	0.100	0.921	0.259	0.800	0.216	0.829	0.112	0.911
3	− 0.432	0.666	1.136	0.256	3.137	**0.002**	1.385	0.166	1.473	0.141
4	3.106	**0.002**	1.674	0.094	2.541	**0.011**	1.825	0.068	− 0.628	0.530
D										
2	− 0.876	0.381	− 1.050	0.294	− 0.555	0.579	− 2.432	**0.015**		
3	− 2.379	**0.017**	− 0.384	0.701	0.616	0.538	0.807	0.420		
4	− 0.508	0.612	− 1.181	0.237	0.336	0.737	2.063	**0.039**		

Significant effects are marked in boldface (p < 0.05). *G + D*—plots sown with grass and diverse forb seed mixture, *D*—plots sown only with diverse forb seed mixture.

Weed cover and species richness decreased significantly with time in both G + D and D plots. In G + D plots weed cover was significantly higher in G + D0 than in G + D1 plots in the first two years. The weed species richness was similar in all study years between the G + D treatments. Weed species cover and richness were in general similar in D plots; only in the second study year were both variables higher in D0 plots than D1 plots.

The cover of *Festuca pseudovina* did not change with time in G + D plots and only differed in the third study year, when it was higher in G + D1 than G + D0 treatments.

### Relation between seed germinability, weather parameters and establishment success

**Figure 4:**
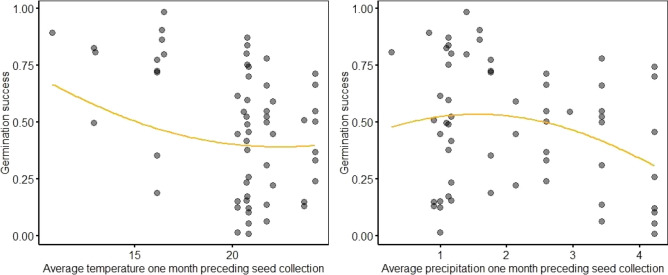
Germination rate of the sown forbs under greenhouse conditions in relation to 1 month average temperature and precipitation preceding seed collection.

Germination rate of the sown forbs in greenhouse differed significantly between the years, being highest in 2015 (Supplementary Tables S5, S6). We did not find any effect of seed mass on germination rate of sown forbs in greenhouse (ß = − 1.296, SE = 1.524, z = − 0.8502, p = 0.3953). Temperature and precipitation conditions before seed collection had significant effect on germination rate (Table 5, Fig. 4). The germination rate of sown forbs was the highest when the temperature of the month preceding the seed collection was the lowest. A higher number of sown forbs were able to germinate after warmer weather conditions of the month preceding seed collection, although their success showed great variability. Germination rate was lower at higher average precipitation in the month preceding seed collection.

**Table 5:** Effect of weather parameters (one-month average temperature and precipitation) preceding seed collection on germination rate of the sown forbs in greenhouse.

Weather effect on germination	P (x)	P (x^2^)	Pseudo R^2^
Average temperature 1 month preceding seed collection	0.0015	0.2550	0.1591
Average precipitation 1 month preceding seed collection	0.0384	0.1176	0.1076

P (x) and P (x^2^) are the P-values for the original and polynomial (quadratic) variable, respectively; pseudo R^2^ is the squared correlation of predictor and link-transformed response from the model containing only the given predictor.

Germination rate of the sown forbs under greenhouse conditions and their cover in field in the year following sowing in general were not related (Table 6).

**Table 6:** Relation of germination rate in greenhouse and year of collection of the sown forbs to their cover in field in the year following sowing (GLM).

Germination and year effect on cover	G + D		D	
	z	p	z	p
Germination rate	− 3.524	** < 0.001**	0.333	0.739
Year 2015	− 8.068	** < 0.001**	8.296	** < 0.001**
Year 2016	− 12.877	** < 0.001**	− 9.172	** < 0.001**
Year 2017	− 13.692	** < 0.001**	− 10.943	** < 0.001**
Germination rate: year 2015	0.703	0.488	− 7.787	** < 0.001**
Germination rate: year 2016	1.849	0.064	− 0.478	0.632
Germination rate: year 2017	1.305	0.192	− 1.170	0.242

Significant effects are marked in boldface (p < 0.05). *G + D*—plots sown with grass and diverse forb seed mixture, *D*—plots sown only with diverse forb seed mixture.

Seed mass of the sown forbs had significant positive effect on their cover in field in the year following sowing; higher seed mass resulted in higher cover of the sown forbs (Table 7).

**Table 7:** Relation of the seed mass of the sown forbs to their cover in field (GLM).

Seed mass and year effect on coverage	G + D		D	
	z	p	z	p
Seed mass	9.544	** < 0.001**	7.431	** < 0.001**
Year 2015	− 7.540	** < 0.001**	9.007	** < 0.001**
Year 2016	− 13.574	** < 0.001**	− 8.917	** < 0.001**
Year 2017	− 14.208	** < 0.001**	− 11.218	** < 0.001**
Seed mass: year 2015	− 7.538	** < 0.001**	− 0.643	0.520
Seed mass: year 2016	− 10.608	** < 0.001**	− 4.616	** < 0.001**
Seed mass: year 2017	− 9.880	** < 0.001**	− 5.724	** < 0.001**

Significant effects are marked in boldface (p < 0.05). *G + D*—plots sown with grass and diverse forb seed mixture, *D*—plots sown only with diverse forb seed mixture.

## Discussion

Our results highlight the importance to introduce grasses and forbs at the same time to the target area to achieve the targeted high diversity of the restored grassland. Later introduction of forbs is usually less successful, but still costly, as the forbs cannot establish without the severe disturbance of entire removal of the existing vegetation.

In our study, both seed sowings, all the tested treatments applied alone or combined, resulted in the development of a high cover of grassland species within a short time period. However sown forb species were more successful in fallow plots, which supports our first hypothesis. In plots where *F. pseudovina* was sown prior to forbs both the cover and richness of sown forbs were lower, than in fallow plots, especially in the second study year. This indicates that competition among forb species is lower than competition between forbs and grasses. The sown grasses can hamper the development of other species groups in different stages of establishment, some of them already in the germination stage by their expressed allelopathic phytochemicals^43–45^. Furthermore, due to their high competitiveness that arises from their high allocation in belowground biomass (i.e., root system and clonal organs), efficient resource use ability and vegetative spreading ability, grasses can easily dominate grassland communities^46,47^. Besides, fast-growing grasses also increase competition for light^48^ and create microsite limitation^24^, that hampers the establishment of new-coming species.

In our system, *F. pseudovina* not only decreased the success of sown forbs but also that of weeds. *F. pseudovina* had especially negative effect on weeds in combination with forb species, supporting our second hypothesis, namely that both species groups hamper weed encroachment. As cover of weeds decreased over the years, they were replaced by sown forb species in plots where both sowing treatments (G + D) were applied; the grass cover was stable between the years, but that of sown forbs increased at the expense of weeds. This finding is in accordance with Werner et al.^33^ in general weeds are poor competitors compared to the sown species, both grasses and forbs, and are displaced by the latter species groups. The strong negative effect of the combination of the grass and diverse forb seed mixture is also supported by Humphries et al.^22^, who found that strong competition by other species is the most successful way to decrease weeds abundance.

As we hypothesized, time since grass sowing and cropland abandonment had a negative effect on the establishment success of sown forb species. Sown forb species were the most successful in the young grass-matrix or fallows, where competition by other species groups was still low and resources were not restricted. We also observed that changes in their cover mostly occurred from the first year to the second; the first year was important for forbs to establish and they increased their cover starting from the second year. The four-year long observations also support the importance of sowing forbs simultaneously with grasses or with just a one-year delay.

Grasses expressed a strong priority effect, so that the later introduced forb species were less successful. This is in accordance with other studies, where similarly to our study it was found, that grasses hamper the establishment of later-arriving species^33,41^ and prevent the development of species-rich grassland^29^. The previous studies found that even a short time difference in sowing grasses and forbs gives advantage for grasses, but in our study, we found that forbs can be still successful after one-year delay. A time lag longer than one year resulted in an irreversible advantage for *F. pseudovina*, as negative effects of grasses probably accumulated over the years (resource limitation, plant-soil feedback). To create and maintain a high diversity grassland, forbs, especially rare and weak competitors, should be sown as soon as possible, as even so there is high chance for some of them to perish over the years^33,38^. Besides, further interventions (harrowing, grazing) may also be needed to create suitable microhabitats for later-arriving species^35,36^.

Not only grass sowing, but also weed encroachment in fallow plots delayed sown forbs establishment. We observed that a two- or three-year-long fallow period resulted in a higher success of weeds, due to the lack of competitors and continuous seed-rain resulting the accumulation of a dense soil seed bank^23,29,49,50^, enabling them to dominate the unsown plots. We also detected a decreasing cover and richness of weeds after sowing the forb species. This is in accordance with other studies^22,24,51^, which found that restoration practices including propagule addition and follow-up management by mowing, can suppress weedy vegetation. It was however surprising that sowing in one-year-old fallows resulted in higher cover of sown forbs than sowing immediately after abandonment. This may be related to a higher overall seed germinability in that particular year, as seeds collected for sowing in one-year-old fallows (2015) also had the highest germination rates in the greenhouse. The difference was not observable in grass-matrix plots, suggesting that grass-competition had the strongest effect on the community development of those plots.

In general germination rate was not related to cover. One reason for this may be that under field conditions a high intraspecific competition was present between the individuals of species with high germination rate and negative frequency-dependence resulted in a decreased survival rate of individuals^52^. Another reason can be that in the field unfavourable post-germination conditions resulted in high mortality of seedlings, so the establishment of species with lower germination rate and gradual emergence may have been more successful^42,53,54^.

In our study the establishment success of sown forbs expressed by cover was positively related with seed mass; larger seed mass resulted in higher cover^55^. Larger-seeded species have an advantage at the early stages of their life cycle, as the resources for growing are provided for seedlings, so they can escape unfavourable conditions^56^. In natural or semi-natural communities, species with small seed would compensate with a higher seed number^56–59^ but our study design could not capture this trade-off, as we used equal seed numbers for all species. Seed dormancy can also contribute to the lower cover of small-seeded species, as small-seeded species more often have persistent seeds than species with larger seeds^60^.

Seed germinability was species-specific and was affected both by temperature and precipitation preceding seed collection. According to Fenner^42^, there is not an exact recipe based on which we can predict seed germinability. Weather parameters experienced by the seeds during their maturation can have contrasting effects on different species. High temperature in general decreases seed dormancy and increases seed germinability, but also results in thicker seed coat which in turn reduces seed germinability. In our study seed collection dates ranged from May to September, during which period temperature changed considerably. The highest germination rates were observed after seeds experienced lower temperatures during maturation; however, this was characteristic only for a few species, probably whose seed maturation ends in late spring or early summer. The majority of seeds germinated after being exposed to higher temperatures during their maturation, although higher temperature reduced overall germination rates. Lower germination rate does not necessarily equal with lower viability^59^; germination rate can be lower as a result of temperature-induced seed dormancy^42^. Similar to the effects of temperature, depending on the mechanism behind dormancy, drought can have similar contrasting and species-specific effects on seeds^42^. In our study the responses to precipitation preceding seed collection showed a high variation. Most of the seeds germinated better after drier conditions, presumably after experiencing summer drought, indicating the presence of autumn-germinating species and seeds. With increasing average precipitation, the germination rate slightly decreased but remained relatively high. Dormancy as well as other factors, like seed degradation and other unmeasured environmental parameters can be a cause of decreasing germination rate.

Based on our results, in the upcoming grassland restoration projects aiming to restore high-diversity grasslands we suggest the introduction of the target plant species simultaneously to the target area. This would reduce the necessity of severe disturbances (i.e., sward removal) later. However, because of the high variation in species germinability between years, reseeding may be still required. An overseeding with a one-year time lag should still result in high establishment success of the target forb species despite the presence of established grasses and still would minimize later costs.

## Methods

### Study site and experimental design

The study was conducted in an experimental site in Hajdúdorog (47.825718, 21.494500), East-Hungary. The climate of the region is continental, the mean annual temperature is 10.0 °C and the mean annual precipitation is 545 mm^61^. The experimental site is a private garden that is a former agricultural land. Agricultural management was stopped in 2014, prior to the start of the experiment. The last cultivated crop was potato. No remnants of natural- or semi-natural grasslands are present adjacent to the study site. We established 36 experimental plots of 4 m × 4 m in October 2014 (Fig. 1, Supplementary Fig. S5). Plots were arranged in a regular grid, with treatments assigned to plots in a regular pattern. One-meter-wide buffer zones were designated between plots. Plots were prepared by ploughing, rotary hoeing and raking, to remove vegetation remnants and roots and to create a fine-textured seed bed for seed sowing. Seed sowing took place yearly in October (21 Oct 2014, 22 Oct 2015, 14 Oct 2016, 11 Oct 2017). Plots received grass (G) seeds (*Festuca pseudovina*), diverse forb seed mixture (D mixture) or the combination of both (G + D). In all treatments with G, grasses were sown in 2014 (Year 0). Sowing D mixture had four levels: sowing in Year 0, 1, 2 or 3, i.e., sowing together with, or 1, 2 or 3 years after the sowing of grass seeds (Table 1). In October 2014 in total 24 plots were sown as follows: (i) in 16 plots only the seeds of *F. pseudovina* (G, G + D1, G + D2, G + D3 plots); (ii) in four plots *F. pseudovina* combined with D mixture (G + D0 plots) and (iii) in four plots only D seed mixture (D0 plots). The remaining 12 plots were left unsown in the first year to regenerate naturally and were managed the same way as the other plot types. The 12 empty (fallow) plots (D1, D2, D3) and 12 of the plots sown with *F. pseudovina* in 2014 (G + D1, G + D2, G + D3) were sown with D seed mixture in the following three years (2015, 2016 and 2017), each in four replicates per year (Table 1). In 2015, 2016 and 2017, prior to the D mixture sowing, G plots to be sown with D mixture were mown, while in unsown fallow plots mowing, rotary hoeing and raking was applied to remove the naturally established vegetation and to create the seed beds before D mixture sowing. In each case seeds were mixed with soil to prevent blowing by wind and to spread seeds more evenly in the plots. We managed the experimental plots by mowing twice in each year, in June and October, right after the vegetation surveys. Mowing was done by a handheld rotary mower, using a cutting height of 10 cm; the mown plant material was raked and removed from the plots immediately. Fertilizers and herbicides were not applied in any of the plots during the whole study period.

Diverse (D) forb seed mixture was composed of twenty forb species characteristic of the loess grasslands of the region (Supplementary Table S7). We collected the seeds from regional populations in the summer-autumn of each sowing year, at the peak of seed maturation period of each species. Seed collection followed the ENSCONET protocol^62^. Seeds were collected outside protected areas and none of the used species is under legal protection in Hungary. Therefore, no permissions were required for seed collection. Seeds were stored dry and cleaned manually. *F. pseudovina* was sown in a density of 20 kg/ha, which is the typical sowing density in dry grasslands in the region^31^. Forb species were represented in equal density in the D mixture with 1000 seeds each sown per the 16-m^2^-sized plots. The total amount of the grasses was 84% of the seeds sown in the G + D mixture, which corresponds to the proportion used in the restoration practice in the study region^27^.

### Vegetation sampling

Between 2015 and 2019, percentage cover of all vascular plant species in plots where sowing previously occurred was recorded in June and October in four 1 m × 1 m-sized permanent subplots per plot (in total 144 plots). As species overlapped, total cover scores in a subplot can exceed a hundred-percent. Subplots were established inside each plot with 0.5-m-wide buffer zones from the plot edges and 1-m-wide buffers between the subplots. Survey of each plot started one year after sowing; i.e., G, D0 and G + D0 plots were sampled from 2015; D1 and G + D1 plots from 2016, D2 and G + D2 plots from 2017, and D3 and G + D3 plots from 2018, respectively.

To test whether there were differences between the germination rate of the collected seeds in the four years, a germination test of the sown forb species was conducted each year. This enabled us to compare the germination rate in the greenhouse and establishment capacity in the field for each sown forb species. Seed mass of each sown forb species was measured for three lots of 100 seeds with an analytical balance prior to seed sowing in each year in October. The measured seeds were sown in October (at the same time when the seeds were sown in the field experiment) in sterilized soil in plastic pots and kept in unheated greenhouse. Pots were watered every other day with tap water. The emerged seedlings were counted and removed weekly until no new seedlings were detected for three weeks.

### Data processing and analysis

We classified species into three functional groups: (i) matrix grass (*F. pseudovina*), (ii) sown forb species (20 species, see Supplementary Table S7) and (iii) weeds. Weeds were classified based on Borhidi social behaviour types^63^; weeds (e.g. *Capsella bursa-pastoris*), ruderal competitors (e.g. *Taraxacum officinale*), adventive competitors (e.g. *Ambrosia artemisiifolia*) and introduced alien species (e.g. *Anethum graveolens*) were considered as weeds. Nomenclature follows Király^64^. We detected in total 40 other species (non-weed, non-sown) with an average cover of 16.13%. Given their low cover, we did not consider these species in the analyses of the functional groups. From cover data recorded in June and October of the same year, the higher one was used for each species. We retrieved average daily precipitation and temperature data from the database of the Hungarian Meteorological Service; from a 30-day-period before the seed collection dates of each forb species in each year.

In our analyses to study vegetation development between all treatment types we used the data from the first two years following the final sowing event of the given treatment. We included the cover data from G + D1 and G + D2 plots in the analysis of G plots, as before sowing D mixture into them, they served as G plots. To study the success of sown forbs in longer time period than two years, we used the four-year long data available from plots D0, G + D0, D1 and G + D1.

All data handling and statistical data analyses were carried out in R (v. 4.0.5, R Core Team)^65^. Throughout our analyses we mainly used generalised linear regression models (GLM) with the “glmmTMB” R-package^66^. Estimated marginal means (EMM) and factor-level comparisons were acquired with the R-package “emmeans”^67^. Initially plot ID was used as random factor to control for the non-independence of the replicates, but we excluded them from the final models, because of the very small random intercept variances, often leading to model convergence warnings or errors.

To test the effect of treatment and development time on species cover and richness, we used log-linked Gamma and quasi-Poisson generalized linear regression models (GLMs), respectively. In these models predictors were study year (as categorical factor, levels: 1, 2) and treatment type, also with control to their interaction. As response variables, we tested species cover and richness of sown forbs and weeds, and cover of *Festuca pseudovina*.

To test how species cover and richness changed across years in the separate treatments [i.e., with the age of grass-matrix (G + D) and fallow (D)], again log-linked Gamma and quasi-Poisson GLMs were fitted, respectively. In these models, the predictor was the age of grass-matrix/fallow (levels: 0–3) as categorical factor. Response variables were cover and richness of sown forbs and weeds, and cover of *Festuca pseudovina*.

Furthermore, we assessed the effect of development time on the difference between G + D0 and G + D1, and between D0 and D1 treatments (i.e., delayed sowing with one year), in species cover (log-linked Gamma GLM) and richness (quasi-Poisson GLM), by specifying study year (1–4, as categorical factor) and treatment type as predictors, and also controlling for their interaction. Hence we were able to assess for each study year whether or not there were significant differences between G + D0 and G + D1 plots, and between D0 and D1 plots.

We tested how average germination rate changed across survey years, by fitting a Beta GLMM, with germination rate as response, year (as categorical factor, levels: 2014–2017) as predictor, and species as random factor. We also tested the effect of seed mass on germination rate in a separate model, using only seed mass as predictor and species as random factor. In additional models, we assessed the effect of weather parameters [average temperature (°C) and rainfall (mm)] preceding seed collection on germination rate. We used separate models for temperature and rainfall to avoid multicollinearity. In the models we specified the orthogonal polynomials of the original weather variables to the power of 1 and 2, in order to be able to potentially detect non-linear effect of the utilized weather parameters. In all Beta models, prior to model fitting germination rate was transformed because Beta GLM(M)s generally cannot handle data containing zeros or ones:

$$y{^{\prime}}=\frac{y\left(N-1\right)+0.5}{N}$$

where y and y′ are the original and transformed variables (germination proportion), respectively, and N is the number of values in the variable y (number of seeds, i.e. 100 for each species).

## Supplementary Information

Supplementary Information.

## Data Availability

The datasets generated during and/or analysed during the current study are available from the corresponding author on reasonable request.
