# Supplementary material for: Co-seeding grasses and forbs supports restoration of species-rich grasslands and improves weed control in ex-arable land
Source: Sci Rep. 2022 Dec 8;12:21239. doi: 10.1038/s41598-022-25837-4 (PMC9732298; doi:10.1038/s41598-022-25837-4)

**Supplementary Table S1**. Differences of species groups cover and richness between first and second study years within grass-matrix and fallows age groups (factor-level comparisons with emmeans). Significant differences are marked in boldface (p<0.05). Abbreviations: G+D-plots sown with grass and diverse forb seed mixture; D-plots sown only with diverse forb seed mixtures. Numbers indicate the age of the grass-matrix (G+D plots) or fallows (D plots) when diverse forb seed mixture was sown in the plots.

|  | |  | Sown forbs | | | | Weed | | | | | | *Festuca pseudovina* | |
| --- | --- | --- | --- | --- | --- | --- | --- | --- | --- | --- | --- | --- | --- | --- |
|  |  | | *Cover (%)* | | *Richness* | | | *Cover (%)* | | | *Richness* | | *Cover (%)* | |
| Plot type | | Study years | t | p | t | p |  | |  | t | | p | t | p |
| G+D0 | | 1-2 | -2.940 | **0.004** | 0.067 | 0.947 | 5.735 | | **<0.001** | 6.400 | | **<0.001** | 0.551 | 0.583 |
| G+D1 | | 1-2 | -3.344 | **0.001** | -0.074 | 0.941 | 5.417 | | **<0.001** | 5.544 | | **<0.001** | 0.330 | 0.742 |
| G+D2 | | 1-2 | 2.101 | **0.038** | -0.333 | 0.740 | 1.566 | | 0.120 | -0.457 | | 0.648 | -0.713 | 0.477 |
| G+D3 | | 1-2 | -0.495 | 0.622 | -0.648 | 0.518 | -2.573 | | **0.011** | -1.208 | | 0.229 | 0.041 | 0.967 |
| D0 | | 1-2 | -4.601 | **<0.001** | -1.791 | 0.076 | 4.171 | | **<0.001** | 3.222 | | **0.002** |  |  |
| D1 | | 1-2 | -3.829 | **<0.001** | -0.479 | 0.633 | 5.429 | | **<0.001** | 5.695 | | **<0.001** |  |  |
| D2 | | 1-2 | -7.400 | **<0.001** | -2.651 | **0.009** | 2.928 | | **0.004** | 0.496 | | 0.621 |  |  |
| D3 | | 1-2 | -8.582 | **<0.001** | -1.150 | 0.252 | -1.725 | | 0.087 | -0.545 | | 0.587 |  |  |

**Supplementary Figure S1**. Differences of species groups cover and richness between first and second study years within grass-matrix and fallows age groups. Lower-case letters indicate significant differences between the two study years within grass-matrix (G+D plots) and fallows (D plots) age groups (factor-level comparisons with emmeans, p<0.05). Boxplot lines represent median values. Abbreviations: G+D-plots sown with grass and diverse forb seed mixture; D-plots sown only with diverse forb seed mixtures. Numbers indicate the age of the grass-matrix (G+D plots) or fallows (D plots) when diverse forb seed mixture was sown in the plots. Symbols:
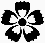
- sown forb species;
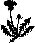
- weeds;
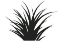
- *Festuca pseudovina.*


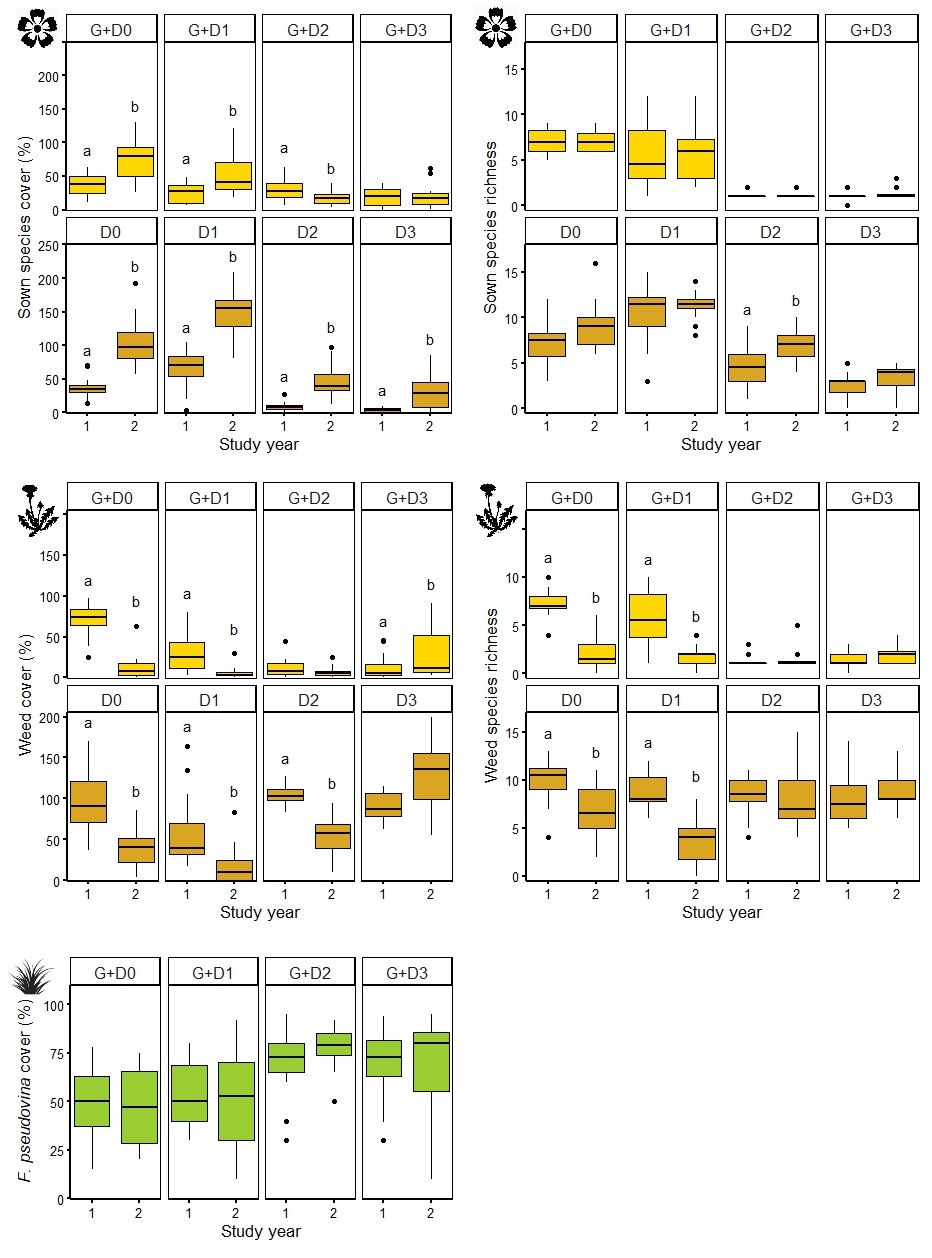


**Supplementary Table S2**. Differences between grass-matrix ages and fallow ages in species-groups cover and richness in the two study years pooled together (overall) and in the two study years separately (factor-level comparisons with emmeans). Significant differences are marked in boldface (p<0.05). Abbreviations: G+D-plots sown with grass and diverse forb seed mixture; D-diverse forb seed mixture. Numbers indicate the age of the grass-matrix (G+D plots) or fallows (D plots) when diverse forb seed mixture was sown in the plots.

|  | | Sown forbs | | | | | | | Weed | | | | | | | | | | | *Festuca pseudovina* | | | | | |
| --- | --- | --- | --- | --- | --- | --- | --- | --- | --- | --- | --- | --- | --- | --- | --- | --- | --- | --- | --- | --- | --- | --- | --- | --- | --- |
|  | | *Cover (%)* | | | *Richness* | | | | | | | *Cover (%)* | | | *Richness* | | | | | | *Cover (%)* | | | |  |
| Plot types | t | | p | | t | | | p | | t | | | p | | | | t | p | | | | t | | p | |
| Overall | | | | | | | | | | | | | | | | | | | | | | | | | |
| G+D0-G+D1 | | 2.541 | | 0.059 | | 2.076 | 0.167 | | | | 3.731 | | | **0.002** | | 1.362 | | | 0.526 | | | -0.870 | 0.820 | | |
| G+D0-G+D2 | | 5.397 | | **<0.001** | | 10.204 | **<0.001** | | | | 5.672 | | | **<0.001** | | 5.693 | | | **<0.001** | | | -4.436 | **<0.001** | | |
| G+D0-G+D3 | | 6.608 | | **<0.001** | | 10.124 | **<0.001** | | | | 2.150 | | | 0.144 | | 4.753 | | | **<0.001** | | | -3.693 | **0.002** | | |
| G+D1-G+D2 | | 2.857 | | **0.026** | | 8.915 | **<0.001** | | | | 1.941 | | | 0.217 | | 4.454 | | | **<0.001** | | | -3.565 | **0.003** | | |
| G+D1-G+D3 | | 4.068 | | **<0.001** | | 8.808 | **<0.001** | | | | -1.582 | | | 0.393 | | 3.441 | | | **0.004** | | | -2.823 | **0.028** | | |
| G+D2-G+D3 | | 1.211 | | 0.621 | | -0.214 | 0.997 | | | | -3.522 | | | **0.003** | | -1.146 | | | 0.662 | | | 0.743 | 0.880 | | |
| D0-D1 | | -2.935 | | **0.021** | | -3.757 | **0.002** | | | | 4.372 | | | **<0.001** | | 3.697 | | | **0.002** | | |  |  | | |
| D0-D2 | | 7.141 | | **<0.001** | | 3.820 | **0.001** | | | | -1.335 | | | 0.543 | | 0.079 | | | 1.000 | | |  |  | | |
| D0-D3 | | 10.762 | | **<0.001** | | 8.604 | **<0.001** | | | | -3.571 | | | **0.003** | | -0.480 | | | 0.963 | | |  |  | | |
| D1-D2 | | 10.077 | | **<0.001** | | 7.375 | **<0.001** | | | | -5.707 | | | **<0.001** | | -3.637 | | | **0.002** | | |  |  | | |
| D1-D3 | | 13.697 | | **<0.001** | | 11.522 | **<0.001** | | | | -7.943 | | | **<0.001** | | -4.164 | | | **<0.001** | | |  |  | | |
| D2-D3 | | 3.621 | | **0.002** | | 5.240 | **<0.001** | | | | -2.236 | | | 0.120 | | -0.562 | | | 0.943 | | |  |  | | |
| Year 1 | | | | | | | | | | | | | | | | | | | | | | | | | |
| G+D0-G+D1 | | 1.999 | | 0.194 | | 1.537 | 0.419 | | | | 2.797 | | | **0.030** | | 1.668 | | | 0.345 | | | -0.505 | 0.958 | | |
| G+D0-G+D2 | | 1.297 | | 0.567 | | 7.281 | **<0.001** | | | | 6.095 | | | **<0.001** | | 7.220 | | | **<0.001** | | | -2.505 | 0.064 | | |
| G+D0-G+D3 | | 3.451 | | **0.004** | | 7.281 | **<0.001** | | | | 5.674 | | | **<0.001** | | 7.045 | | | **<0.001** | | | -2.356 | 0.091 | | |
| G+D1-G+D2 | | -0.702 | | 0.896 | | 6.349 | **<0.001** | | | | 3.297 | | | **0.007** | | 6.135 | | | **<0.001** | | | -2.000 | 0.194 | | |
| G+D1-G+D3 | | 1.452 | | 0.470 | | 6.350 | **<0.001** | | | | 2.876 | | | **0.024** | | 5.893 | | | **<0.001** | | | -1.852 | 0.255 | | |
| G+D2-G+D3 | | 2.154 | | 0.142 | | 0.001 | 1.000 | | | | -0.421 | | | 0.975 | | -0.457 | | | 0.968 | | | 0.148 | 0.999 | | |
| D0-D1 | | -2.461 | | 0.071 | | -3.279 | **0.007** | | | | 2.463 | | | 0.071 | | 1.094 | | | 0.694 | | |  |  | | |
| D0-D2 | | 6.449 | | **<0.001** | | 3.100 | **0.013** | | | | -0.323 | | | 0.988 | | 1.514 | | | 0.432 | | |  |  | | |
| D0-D3 | | 9.601 | | **<0.001** | | 5.807 | **<0.001** | | | | 0.423 | | | 0.974 | | 1.636 | | | 0.362 | | |  |  | | |
| D1-D2 | | 8.910 | | **<0.001** | | 6.135 | **<0.001** | | | | -2.785 | | | **0.031** | | 0.422 | | | 0.975 | | |  |  | | |
| D1-D3 | | 12.062 | | **<0.001** | | 8.309 | **<0.001** | | | | -2.040 | | | 0.179 | | 0.545 | | | 0.948 | | |  |  | | |
| D2-D3 | | 3.152 | | **0.011** | | 3.058 | **0.014** | | | | 0.746 | | | 0.878 | | 0.123 | | | 0.999 | | |  |  | | |
| Year 2 | | | | | | | | | | | | | | | | | | | | | | | | | |
| G+D0-G+D1 | | 1.594 | | 0.386 | | 1.398 | 0.503 | | | | 2.480 | | | 0.068 | | 0.650 | | | 0.915 | | | -0.726 | 0.887 | | |
| G+D0-G+D2 | | 6.337 | | **<0.001** | | 7.150 | **<0.001** | | | | 1.926 | | | 0.223 | | 1.208 | | | 0.623 | | | -3.768 | **0.001** | | |
| G+D0-G+D3 | | 5.895 | | **<0.001** | | 7.040 | **<0.001** | | | | -2.634 | | | **0.047** | | -0.001 | | | 1.000 | | | -2.866 | **0.025** | | |
| G+D1-G+D2 | | 4.743 | | **<0.001** | | 6.259 | **<0.001** | | | | -0.553 | | | 0.946 | | 0.565 | | | 0.942 | | | -3.042 | **0.015** | | |
| G+D1-G+D3 | | 4.301 | | **<0.001** | | 6.108 | **<0.001** | | | | -5.113 | | | **<0.001** | | -0.650 | | | 0.915 | | | -2.140 | 0.146 | | |
| G+D2-G+D3 | | -0.442 | | 0.971 | | -0.316 | 0.989 | | | | -4.560 | | | **<0.001** | | -1.208 | | | 0.623 | | | 0.902 | 0.804 | | |
| D0-D1 | | -1.690 | | 0.334 | | -1.990 | 0.198 | | | | 3.721 | | | **0.002** | | 3.756 | | | **0.002** | | |  |  | | |
| D0-D2 | | 3.650 | | **0.002** | | 2.251 | 0.116 | | | | -1.565 | | | 0.402 | | -1.243 | | | 0.601 | | |  |  | | |
| D0-D3 | | 5.619 | | **<0.001** | | 6.418 | **<0.001** | | | | -5.473 | | | **<0.001** | | -2.152 | | | 0.143 | | |  |  | | |
| D1-D2 | | 5.340 | | **<0.001** | | 4.183 | **<0.001** | | | | -5.286 | | | **<0.001** | | -4.889 | | | **<0.001** | | |  |  | | |
| D1-D3 | | 7.309 | | **<0.001** | | 7.990 | **<0.001** | | | | -9.193 | | | **<0.001** | | -5.695 | | | **<0.001** | | |  |  | | |
| D2-D3 | | 1.969 | | 0.206 | | 4.477 | **<0.001** | | | | -3.907 | | | **<0.001** | | -0.918 | | | 0.800 | | |  |  | | |

**Supplementary Figure S2**. Differences between grass-matrix ages and fallow ages in species-groups cover and richness in the two study years. Lower-case letters indicate significant differences of grass-matrix- (G+D plots) and fallow ages (D plots) in the same development stage (factor-level comparisons with emmeans, p<0.05). Boxplot lines represent median values. Abbreviations: G+D-plots sown with grass and diverse forb seed mixture; D-plots sown only with diverse forb seed mixtures. Numbers indicate the age of the grass-matrix (G+D plots) or fallows (D plots) when diverse forb seed mixture was sown in the plots. Symbols:
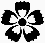
- sown forb species;
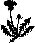
-weeds;
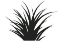
-*Festuca pseudovina.*


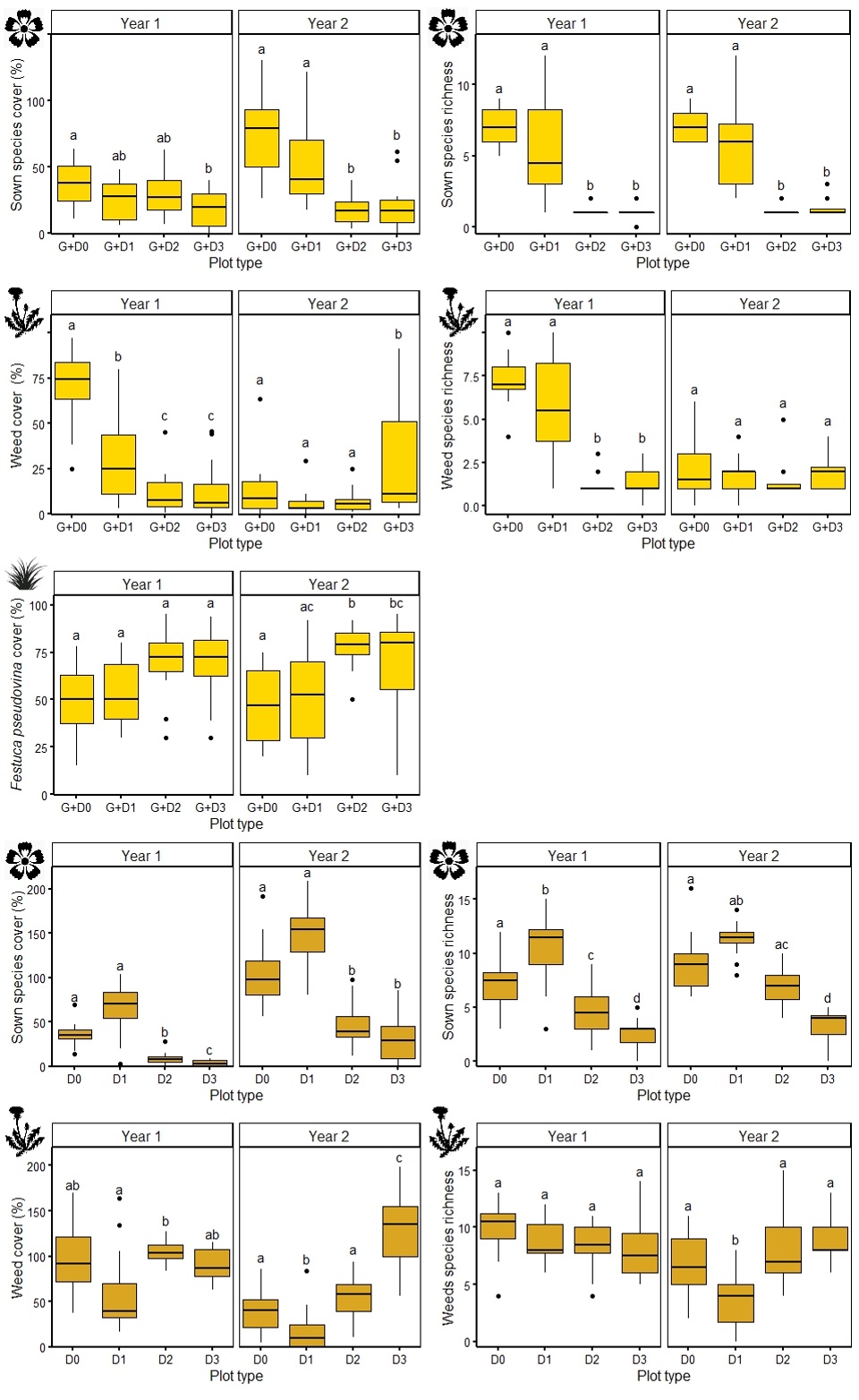


**Supplementary Table S3**. Differences of species groups cover and richness in the first four study years within grass-matrix and fallows age groups (factor-level comparisons with emmeans). Significant differences are marked in boldface (p<0.05). Abbreviations: G+D-plots sown with grass and diverse forb seed mixture; D-plots sown only with diverse forb seed mixtures. Numbers indicate the age of the grass-matrix (G+D plots) or fallows (D plots) when diverse forb seed mixture was sown in the plots.

|  |  | Sown forbs | | | | | | Weed | | | | | *Festuca pseudovina* | | | |
| --- | --- | --- | --- | --- | --- | --- | --- | --- | --- | --- | --- | --- | --- | --- | --- | --- |
|  |  | *Cover (%)* | | *Richness* | | *Cover (%)* | | | | *Richness* | | | | *Cover(%)* | | |
| Plot type | Study year | t | p | t | p | | t | | p | | t | p | | | t | p |
| G+D0 | 1-2 | -3.320 | **0.007** | 0.067 | 0.999 | | 6.622 | | **<0.001** | | 6.400 | **<0.001** | | | 0.394 | 0.979 |
|  | 1-3 | -4.135 | **<0.001** | 0.890 | 0.810 | | 11.880 | | **<0.001** | | 7.271 | **<0.001** | | | 1.340 | 0.540 |
|  | 1-4 | -3.504 | **0.004** | 0.820 | 0.845 | | 9.981 | | **<0.001** | | 6.982 | **<0.001** | | | -0.138 | 0.999 |
|  | 2-3 | -0.815 | 0.847 | 0.8237 | 0.843 | | 5.258 | | **<0.001** | | 1.800 | 0.279 | | | 0.946 | 0.780 |
|  | 2-4 | -0.184 | 0.998 | 0.753 | 0.875 | | 3.359 | | **0.006** | | 1.065 | 0.711 | | | -0.532 | 0.951 |
|  | 3-4 | 0.631 | 0.922 | -0.071 | 1.000 | | -1.899 | | 0.234 | | -0.761 | 0.872 | | | -1.478 | 0.454 |
| G+D1 | 1-2 | -3.812 | **0.001** | -0.074 | 1.000 | | 6.255 | | **<0.001** | | 5.544 | **<0.001** | | | 0.236 | 0.995 |
|  | 1-3 | -3.524 | **0.003** | -0.721 | 0.888 | | 7.443 | | **<0.001** | | 5.808 | **<0.001** | | | -0.743 | 0.880 |
|  | 1-4 | -7.896 | **<0.001** | -1.537 | 0.419 | | 6.387 | | **<0.001** | | 4.992 | **<0.001** | | | 0.751 | 0.876 |
|  | 2-3 | 0.288 | 0.992 | -0.648 | 0.916 | | 1.188 | | 0.636 | | 0.420 | 0.975 | | | -0.979 | 0.762 |
|  | 2-4 | -4.084 | **<0.001** | -1.464 | 0.462 | | 0.132 | | 0.999 | | -0.773 | 0.866 | | | 0.515 | 0.955 |
|  | 3-4 | -4.372 | **<0.001** | -0.820 | 0.845 | | -1.056 | | 0.717 | | -1.187 | 0.636 | | | 1.494 | 0.445 |
| D0 | 1-2 | -7.175 | **<0.001** | -1.791 | 0.283 | | 2.605 | | 0.050 | | 3.222 | **0.009** | | |  |  |
|  | 1-3 | -8.128 | **<0.001** | -0.768 | 0.869 | | 4.282 | | **<0.001** | | 7.316 | **<0.001** | | |  |  |
|  | 1-4 | -7.838 | **<0.001** | -0.453 | 0.969 | | 3.785 | | **<0.001** | | 7.966 | **<0.001** | | |  |  |
|  | 2-3 | -0.953 | 0.776 | 1.028 | 0.733 | | 1.678 | | 0.340 | | 4.614 | **<0.001** | | |  |  |
|  | 2-4 | -0.664 | 0.911 | 1.342 | 0.538 | | 1.180 | | 0.640 | | 5.482 | **<0.001** | | |  |  |
|  | 3-4 | 0.290 | 0.992 | 0.316 | 0.989 | | -0.497 | | 0.960 | | 1.076 | 0.705 | | |  |  |
| D1 | 1-2 | -5.936 | **<0.001** | -0.479 | 0.964 | | 3.390 | | **0.005** | | 5.695 | **<0.001** | | |  |  |
|  | 1-3 | -4.763 | **<0.001** | -0.321 | 0.989 | | 3.411 | | **0.005** | | 6.224 | **<0.001** | | |  |  |
|  | 1-4 | -7.120 | **<0.001** | 1.283 | 0.576 | | 3.310 | | **0.007** | | 5.922 | **<0.001** | | |  |  |
|  | 2-3 | 1.173 | 0.645 | 0.158 | 0.999 | | 0.021 | | 1.000 | | 0.670 | 0.908 | | |  |  |
|  | 2-4 | -1.185 | 0.638 | 1.759 | 0.298 | | -0.080 | | 1.000 | | 0.282 | 0.992 | | |  |  |
|  | 3-4 | -2.357 | 0.091 | 1.602 | 0.382 | | -0.102 | | 1.000 | | -0.388 | 0.980 | | |  |  |

**Supplementary Figure S3**. Differences of species groups cover and richness in the first four study years within grass-matrix and fallows age groups. Lower-case letters indicate significant differences between the study years of the same plot types (factor-level comparisons with emmeans, p<0.05). Boxplot lines represent median values. Abbreviations: G+D-plots sown with grass and diverse forb seed mixture; D-plots sown only with diverse forb seed mixtures. Numbers indicate the age of the grass-matrix (G+D plots) or fallows (D plots) when diverse forb seed mixture was sown in the plots. Symbols:
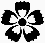
- sown forb species;
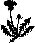
- weeds;
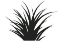
- *Festuca pseudovina.*


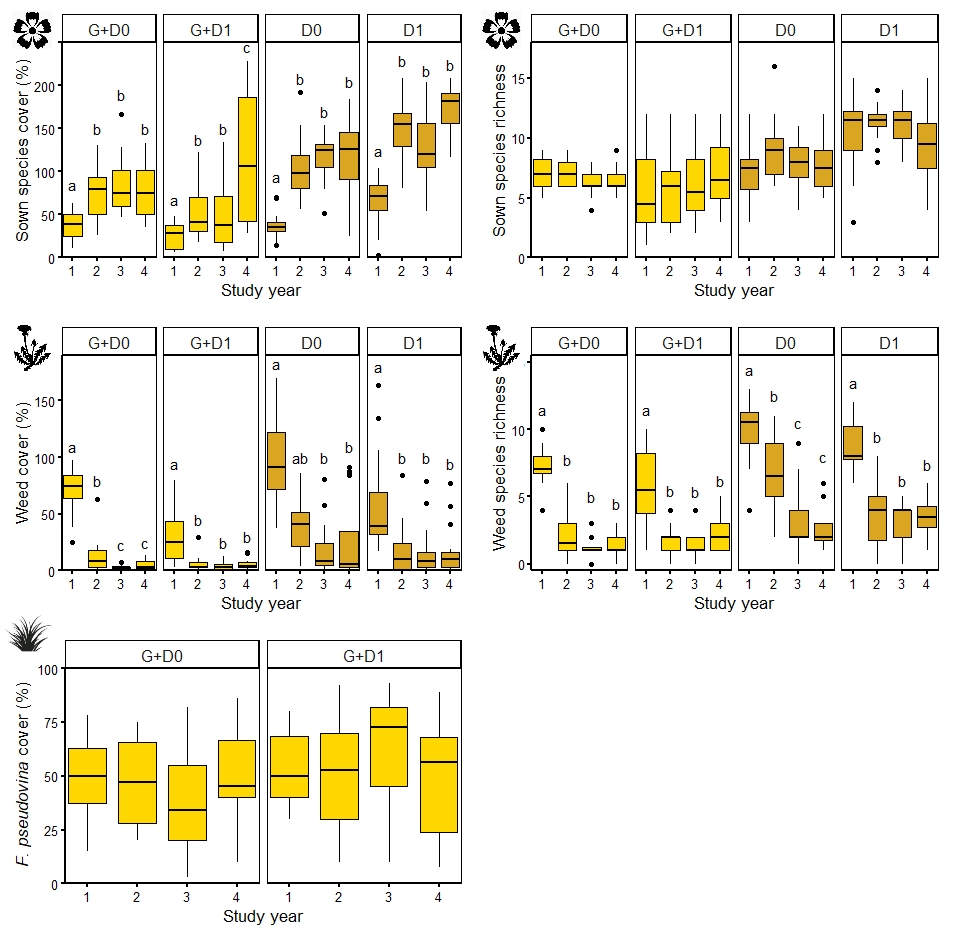


**Supplementary Table S4**. Differences of species groups cover and richness between the two youngest grass-matrixes and fallows in the four study years (factor-level comparisons with emmeans). Significant differences are marked in boldface (p<0.05). Abbreviations: G+D-plots sown with grass and diverse forb seed mixture; D-plots sown only with diverse forb seed mixtures. Numbers indicate the age of the grass-matrix (G+D plots) or fallows (D plots) when diverse forb seed mixture was sown in the plots.

|  |  | Sown forbs | | | | | Weed | | | | | | | *Festuca pseudovina* | | |
| --- | --- | --- | --- | --- | --- | --- | --- | --- | --- | --- | --- | --- | --- | --- | --- | --- |
|  |  | *Cover (%)* | | | *Richness* | | | *Cover (%)* | | | *Richness* | | *Cover (%)* | | | |
| Plot types | Study year | t | p | t | | p | t | | p | t | | p | t | | p | |
| G+D0 - G+D1 | 1 | 2.288 | **0.024** | 1.537 | | 0.127 | 3.230 | | **0.002** | 1.668 | | 0.098 | -0.361 | | | 0.719 |
| G+D0 - G+D1 | 2 | 1.795 | 0.075 | 1.398 | | 0.165 | 2.863 | | **<0.001** | 0.650 | | 0.517 | -0.519 | | | 0.605 |
| G+D0 - G+D1 | 3 | 2.898 | **0.005** | -0.071 | | 0.944 | -1.207 | | 0.230 | -0.761 | | 0.448 | -2.443 | | | **0.016** |
| G+D0 - G+D1 | 4 | -2.105 | **0.037** | -0.820 | | 0.414 | -0.364 | | 0.717 | -1.187 | | 0.238 | 0.528 | | | 0.598 |
| D0 - D1 | 1 | -3.852 | **<0.001** | -3.279 | | **<0.001** | 1.538 | | 0.127 | 1.094 | | 0.275 |  | |  | |
| D0 - D1 | 2 | -2.613 | **0.010** | -1.990 | | **0.049** | 2.323 | | **0.022** | 3.756 | | **<0.001** |  | |  | |
| D0 - D1 | 3 | -0.487 | 0.627 | -2.845 | | **0.005** | 0.667 | | 0.506 | -0.302 | | 0.764 |  | |  | |
| D0 - D1 | 4 | -3.134 | **0.002** | -1.574 | | 0.118 | 1.063 | | 0.290 | -1.753 | | 0.082 |  | |  | |

**Supplementary Figure S4**. Differences of species groups cover and richness between the two youngest grass-matrixes and fallows in the four study years. Lower-case letters indicate significant differences between the two age groups within the four study years of the same plot type (factor-level comparisons with emmeans, p<0.05). Boxplot lines represent median values. Abbreviations: G+D-plots sown with grass and diverse forb seed mixture; D-plots sown only with diverse forb seed mixtures. Numbers indicate the age of the grass-matrix (G+D plots) or fallows (D plots) when diverse forb seed mixture was sown in the plots. Symbols:
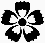
- sown forb species;
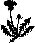
- weeds;
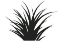
- *Festuca pseudovina*


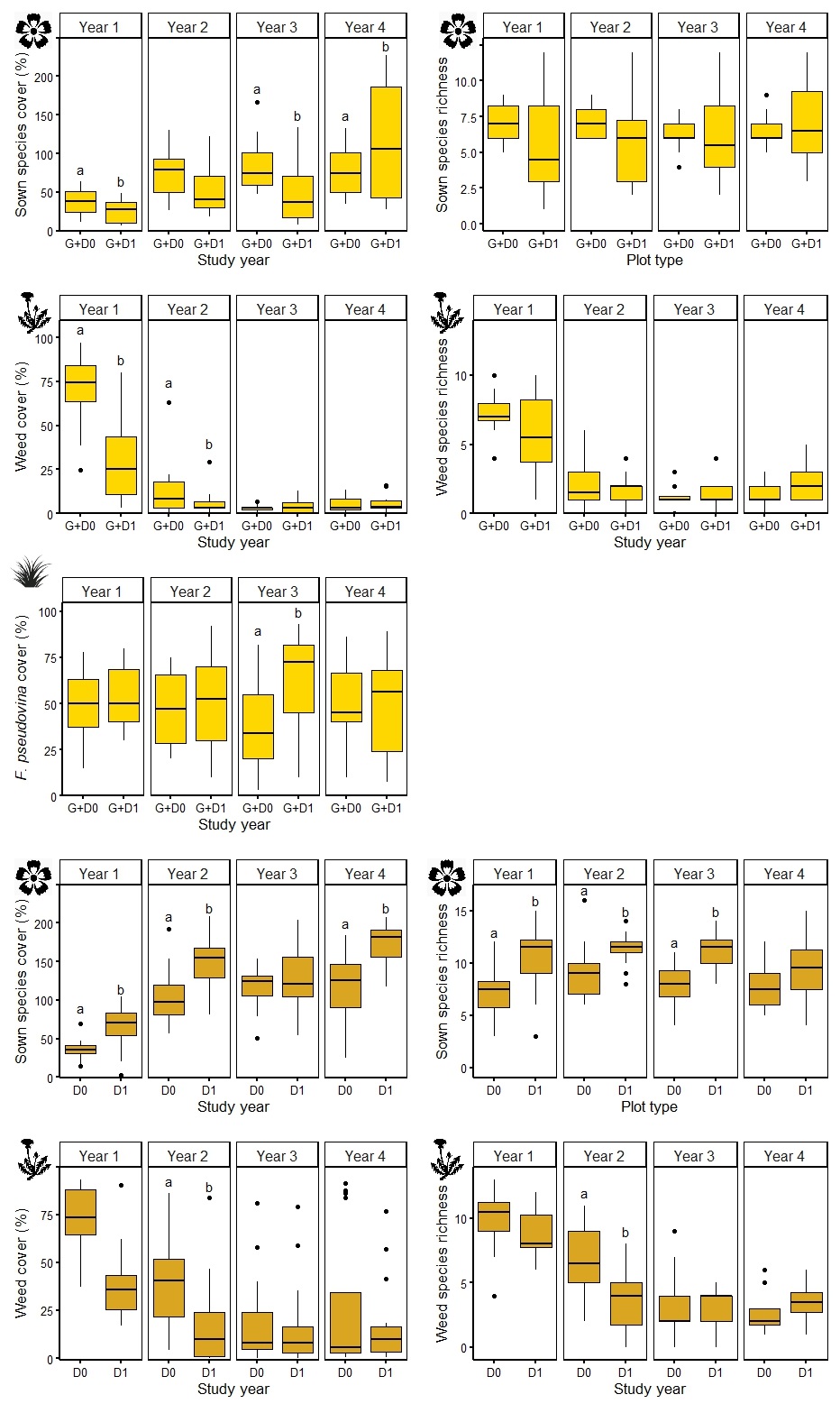


**Supplementary Table S5**. Effect of year of collection on the average germination success of sown forb species under greenhouse conditions (GLMM). Significant differences are marked in boldface (p<0.05).

| Year of collection | z | p |
| --- | --- | --- |
| 2015 | 3.169 | **0.0015** |
| 2016 | -0.359 | 0.7199 |
| 2017 | -1.150 | 0.2501 |

**Supplementary Table S6**. Year to year differences in the average germination success of sown forb species under greenhouse conditions (factor-level comparisons with emmeans). Significant differences are marked in boldface (p<0.05).

| Years | t | p |
| --- | --- | --- |
| 2014-2015 | -3.169 | **0.009** |
| 2014-2016 | 0.359 | 0.984 |
| 2014-2017 | 1.150 | 0.659 |
| 2015-2016 | 3.627 | **0.002** |
| 2015-2017 | 4.492 | **<0.001** |
| 2016-2017 | 0.834 | 0.838 |

**Supplementary Table S7**. Species composition of diverse forb seed mixture, thousand seed weight (mean±sd) of every species and germination capacity (mean±sd) of three lots of 100 seeds under greenhouse conditions, in each collection year.

|  | 1000 seed weight (g) | | | |  | Germination % | | | |
| --- | --- | --- | --- | --- | --- | --- | --- | --- | --- |
|  | 2014 | 2015 | 2016 | 2017 |  | 2014 | 2015 | 2016 | 2017 |
| *Achillea collina* | 0.083±0.003 | 0.073±0.004 | 0.073±0.002 | 0.064±0.007 |  | 80.667±6.028 | 50.333±7.371 | 66.333±9.452 | 43.333±11.060 |
| *Centaurea jacea subsp. angustifolia* | 1.688±0.050 | 1.404±0.081 | 1.418±0.049 | 1.534±0.086 |  | 23.000±4.000 | 23.667±8.505 | 50.000±7.000 | 34.000±5.292 |
| *Cruciata pedemontana* | 0.386±0.019 | 0.512±0.015 | 0.389±0.005 | 0.382±0.008 |  | 49.667±24.542 | 99.333±1.155 | 91.333±3.055 | 98.667±1.155 |
| *Dianthus pontederae* | 0.499±0.027 | 0.493±0.013 | 0.516±0.020 | 0.469±0.008 |  | 73.000±17.436 | 87.667±0.577 | 74.667±10.599 | 83.667±1.528 |
| *Falcaria vulgaris* | 0.988±0.031 | 1.028±0.067 | 0.798±0.054 | 0.771±0.021 |  | 15.000±7.000 | 36.667±3.786 | 30.667±5.033 | 26.667±4.163 |
| *Filipendula vulgaris* | 1.006±0.063 | 1.022±0.037 | 0.758±0.036 | 1.100±0.073 |  | 61.667±7.095 | 84.333±4.619 | 25.333±5.508 | 71.333±2.082 |
| *Galium verum* | 0.313±0.014 | 0.252±0.016 | 0.359±0.006 | 0.264±0.030 |  | 59.333±6.429 | 71.667±10.504 | 78.333±4.726 | 80.000±14.799 |
| *Hypericum perforatum* | 0.068±0.002 | 0.071±0.003 | 0.069±0.002 | 0.055±0.003 |  | 41.667±3.512 | 33.000±1.732 | 52.333±11.504 | 32.333±3.055 |
| *Knautia arvensis* | 3.713±0.030 | 3.322±0.103 | 3.168±0.066 | 3.546±0.081 |  | 52.333±3.215 | 55.000±2.646 | 55.000±3.606 | 53.333±6.351 |
| *Lotus corniculatus* | 1.105±0.031 | 1.018±0.000 | 0.937±0.030 | 1.040±0.011 |  | 21.667±5.774 | 14.333±6.028 | 11.333±1.528 | 27.000±2.646 |
| *Lycopsis arvensis* | 2.679±0.104 | 3.546±0.344 | 2.680±0.137 | 2.699±0.184 |  | 11.667±2.887 | 51.000±10.536 | 5.667±2.309 | 15.000±2.646 |
| *Plantago media* | 0.298±0.006 | 0.249±0.006 | 0.264±0.014 | 0.194±0.013 |  | 44.667±11.846 | 49.000±5.292 | 45.667±4.726 | 17.667±6.110 |
| *Podospermum canum* | 3.799±0.382 | 3.839±0.371 | 3.447±0.151 | 3.006±0.105 |  | 83.000±8.888 | 90.000±1.732 | 81.333±6.028 | 88.333±3.215 |
| *Rapistrum perenne* | 5.407±0.419 | 5.188±0.549 | 6.470±0.454 | 7.345±0.070 |  | 0.667±0.577 | 12.333±3.215 | 4.667±4.041 | 2.667±2.082 |
| *Salvia austriaca* | 1.551±0.113 | 1.789±0.115 | 1.921±0.039 | 1.375±0.130 |  | 35.000±10.536 | 80.333±6.807 | 70.333±4.509 | 35.667±13.204 |
| *Salvia nemorosa* | 0.585±0.018 | 0.675±0.048 | 0.521±0.033 | 0.543±0.044 |  | 18.333±0.577 | 37.667±3.055 | 0.000±0.000 | 0.667±0.577 |
| *Securigera varia* | 4.014±0.111 | 4.928±0.112 | 4.290±0.270 | 3.599±0.100 |  | 14.667±8.021 | 16.667±3.786 | 9.667±3.215 | 3.333±1.528 |
| *Silene viscosa* | 0.211±0.004 | 0.179±0.004 | 0.243±0.007 | 0.194±0.005 |  | 78.000±26.851 | 60.000±12.000 | 87.000±3.000 | 79.000±11.269 |
| *Scabiosa ochroleuca* | 1.284±0.050 | 1.422±0.083 | 1.633±0.108 | 1.630±0.053 |  | 44.667±13.577 | 66.667±11.547 | 54.667±6.658 | 62.667±4.041 |
| *Thymus glabrescens* | 0.144±0.002 | 0.144±0.010 | 0.081±0.011 | 0.054±0.002 |  | 72.333±16.442 | 75.667±1.528 | 13.000±10.817 | 0.333±0.577 |
| Seed density/plot (g) | 29.821±0.136 | 31.154±0.176 | 30.035±0.132 | 29.864±0.072 |  |  |  |  |  |

**Supplementary Figure S5**. Moments of the study: A – The study site in 2014, B – High-diversity seed mixture in 2014, C – Seed bed in a fallow plot prepared for D mixture sowing in 2015, D – vegetation development in a G plot in 2017, E – vegetation development in a G+D1 plot in 2017, F – vegetation development in a G+D3 plot in 2018, G – vegetation development in a D1 plot in 2017, H – vegetation development in a D2 plot in 2017. Phots by Tamás Miglécz.


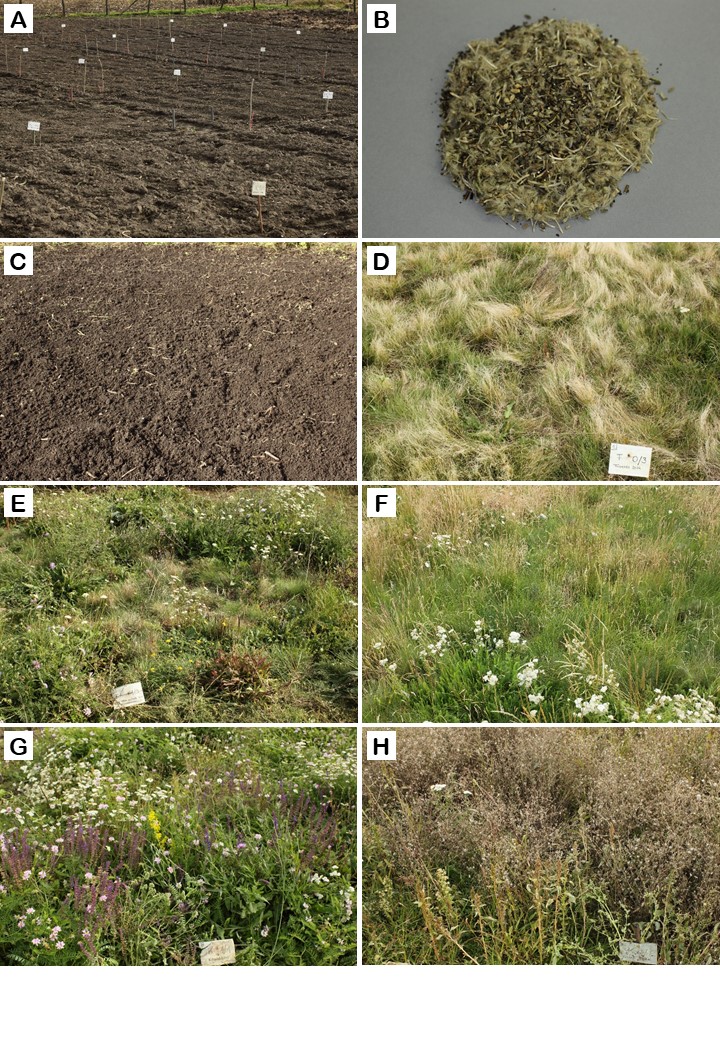

Supplement: Supplementary file 1 — Supplementary Information. [file 41598_2022_25837_MOESM1_ESM.docx]
